# BET inhibitors RVX-208 and PFI-1 reactivate HIV-1 from latency

**DOI:** 10.1038/s41598-017-16816-1

**Published:** 2017-11-30

**Authors:** Panpan Lu, Yinzhong Shen, He Yang, Yanan Wang, Zhengtao Jiang, Xinyi Yang, Yangcheng Zhong, Hanyu Pan, Jianqing Xu, Hongzhou Lu, Huanzhang Zhu

**Affiliations:** 1 State Key Laboratory of Genetic Engineering, Institute of Genetics, School of Life Sciences, Fudan University, Shanghai, 200438 China; 2 Department of Infectious Diseases, and Key Laboratory of Medical Molecular Virology of Ministry of Education/Health, Shanghai Public Health Clinical Center, Fudan University, Shanghai, 200433 China

## Abstract

Persistent latent reservoir in resting CD4+ T cells is a major obstacle in curing HIV-1 infection. Effective strategies for eradication of the HIV-1 reservoir are urgently needed. We report here for the first time that two BET inhibitors, RVX-208, which has entered phase II clinical trials for diverse cardiovascular disorders, and PFI-1, which has been widely studied in oncology, can reactivate HIV-1 from latency. RVX-208 and PFI-1 treatment alone or in combination with other latency reversing agents efficiently reactivated HIV-1 transcription through an up-regulation of P-TEFb by increasing CDK9 Thr-186 phosphorylation in latently infected Jurkat T cells *in vitro*. The two BET inhibitors also reactivated HIV-1 transcription in cART treated patient-derived resting CD4+ T cells *ex vivo*, without influence on global immune cell activation. Our findings, in combination with previous reports, further confirm that BET inhibitors are a group of leading compounds for combating HIV-1 latency for viral eradication.

## Introduction

The introduction of combination antiretroviral therapy (cART) represents a groundbreaking achievement in the effort to combat human immunodeficiency virus-1 (HIV-1) infection, and provides clinicians with a therapeutic opportunity to suppress viral replication and restore the immune function of infected individuals^1,2^. However, long-term cART does not result in HIV-1 eradication because of the presence of long-lived viral reservoirs. cART cessation results in viral rebound within weeks that arises from resting memory CD4+ T cells harboring HIV-1 proviral DNA integrated into the cellular genome^3–5^. Therefore, the development of therapies capable of exhausting this latent viral reservoir, primarily residing within long-lived CD4+ T cells, has become a highly prioritized goal in HIV-1 research.

One approach towards this aim, often referred to as ‘shock and kill’^6^, is characterized by the use of pharmacological agents to reverse HIV-1 latency and turn on the production of viral proteins in latently infected cells, as this would theoretically expose such cells to killing by immune-mediated mechanisms or viral cytopathic effects. A wide range of latency reversing agents (LRAs) has been investigated *in vitro* and *ex vivo* ^7^ with a few candidates being advanced to testing in experimental clinical trials^8–10^. These can be categorized into the following groups, mainly based on pharmacological targets: (1) histone deacetylase inhibitors (HDACi); (2) cytokines and chemokines; (3) DNA methyltransferase inhibitors (DNMTi); (4) histone methyltransferase inhibitors (HMTi); (5) protein kinase C (PKC) activators; (6) positive transcription elongation factor b (P-TEFb) activators; and (7) unclassified agents, such as disulfram^7,11^. However, all these interventions have still not shown any durable decrease in the viral reservoir, and toxicity and target specificity still remain major concerns.

Bromodomains have emerged as attractive candidates for the development of inhibitors targeting gene transcription. Inhibitors of the bromodomain and extra terminal (BET) family recently showed promising activity in diverse disease models^12^. Notably, our and other laboratories have proved that some BET inhibitors including OTX015^13^, JQ1^14,15^ and UMB-136^16^ could induce latent HIV-1 expression in diverse cell culture models and patient-derived resting CD4+ T cells. RVX-208 is a first-in-class, oral BET inhibiror, also known as RVX000222, in development by Resverlogix Corporation (Calgary, AB, Canada) for the treatment of acute coronary syndromes, atherosclerosis^17,18^ and Alzheimer’s disease^19^. This novel small molecule is currently in Phase II clinical trials and the evidence indicates that RVX-208 increases apolipoprotein AI (ApoA-I) and high-density lipoprotein cholesterol (HDL-C) levels as potential therapeutic targets for reducing atherosclerotic disease, in non-human primates and humans^17,20^. The dihydroquinazolinone PFI-1, also named as PF-6405761, has recently been reported as a BET chemical probe derived from optimization of a fragment-screening hit^21^. PFI-1 exhibits activity against cell lines carrying oncogenic rearrangements in the MLL locus. The compound causes significant down-regulation of Aurora B kinase with a decrease in phosphorylation of the Aurora substrate histone 3 S10 (H3S10)^22,23^. The present study explores the ability of the two BET inhibitors to target latent HIV-1 in latently infected Jurkat T cell culture models *in vitro* as well as patient-derived resting CD4+ T cells *ex vivo*. Our results for the first time identify RVX-208 and PFI-1 as potential candidates of anti-HIV-latency therapy.

## Results

### RVX-208 and PFI-1 are potent activator of HIV-1 in different *in vitro* Jurkat T models of latency

**Figure 1:**
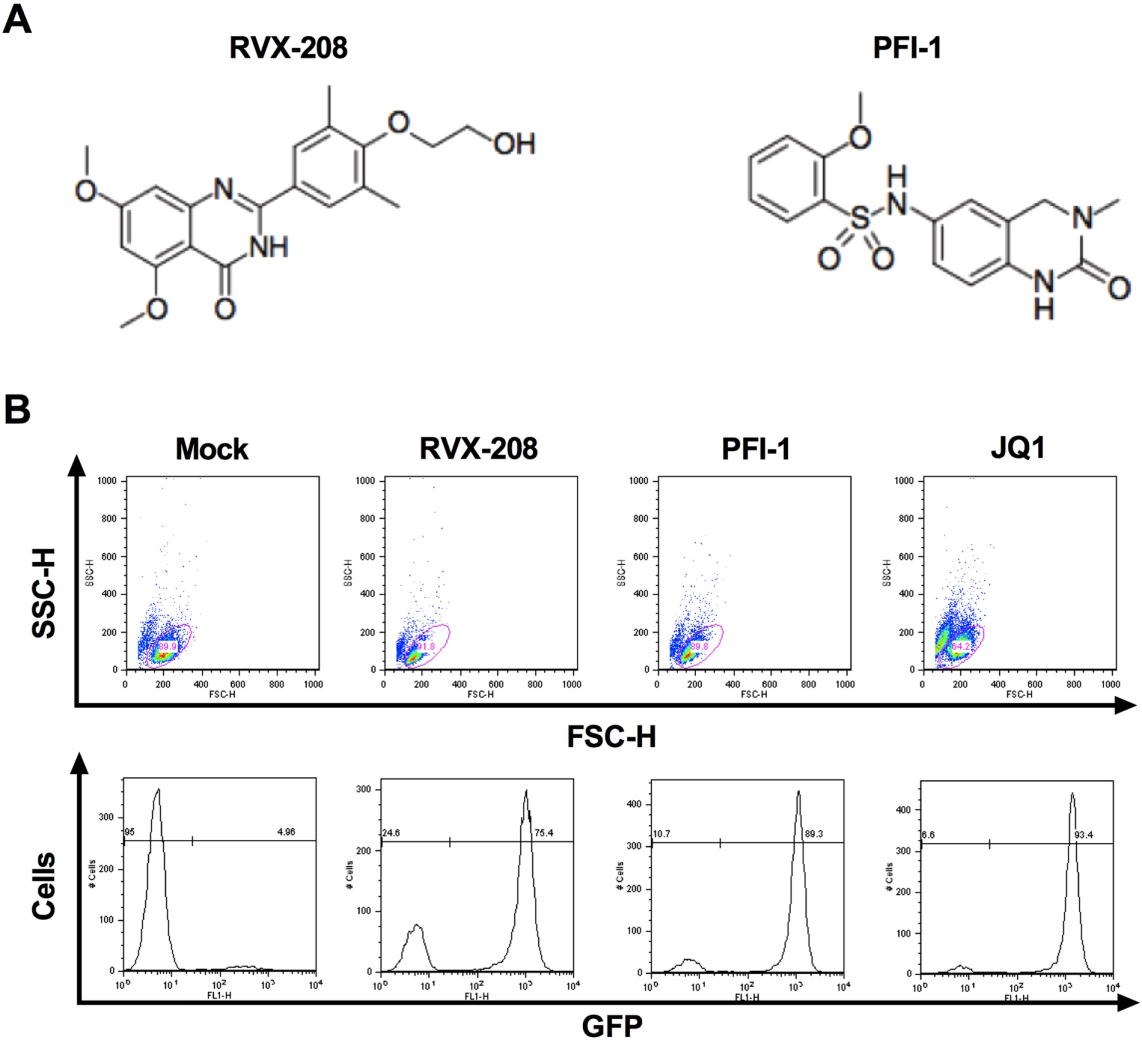
*In vitro* activation of HIV-1 expression by BET inhibitors in Jurkat C11 cell culture models. (**A**) The molecular structure of RVX-208 and PFI-1. (**B**) Induction of GFP expression by 50 μM RVX-208, 5 μM PFI-1 and 1 μM JQ1. Flow cytometry analysis of C11 cells is shown.

**Figure 2:**
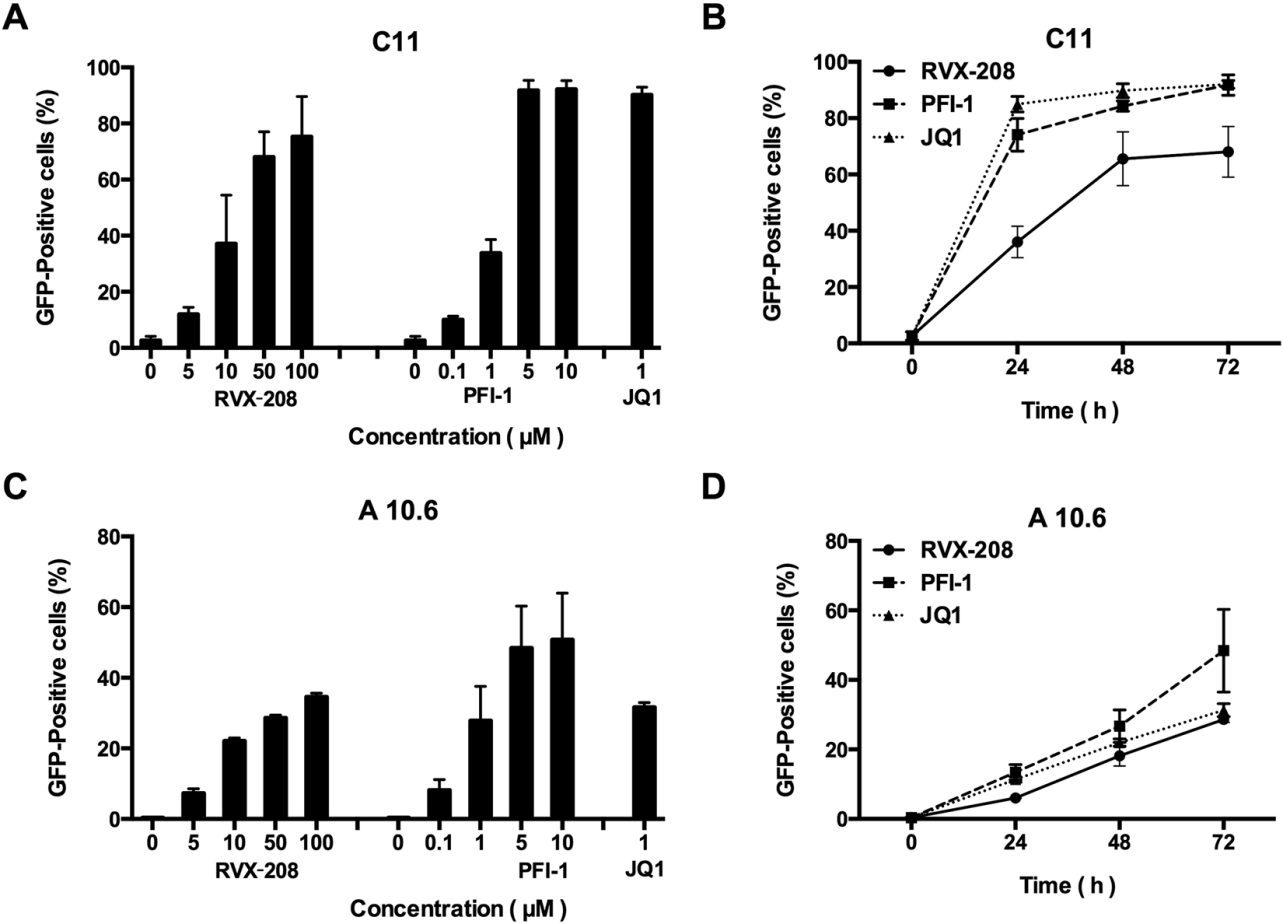
*In vitro* activation of HIV-1 expression by BET inhibitors in diverse Jurkat T cell culture models. (**A** and **C**) A dose response of HIV-1 activation by BET inhibitors was determined by the quantification of GFP reporter activity after a 72-hour treatment in C11 cells (**A**) or A10.6 cells (**C**). (**B** and **D**) Time course of the induction of GFP expression by BET inhibitors. C11 cells (**B**) or A10.6 cells (**D**) were treated with 50 μM RVX-208, 5 μM PFI-1 or 1 μM JQ1 for 24 to 72 hours. Percentage of GFP-positive cells was determined by flow cytometry.

Figure 1A displays the structure of RVX-208 and PFI-1. To determine the effect of RVX-208 and PFI-1 on latent HIV-1 reactivation, we used J-Lat C11 cell line in which transcriptional activation of the latent provirus can be detected in individual cells by flow cytometry, since these cells harbor full-length latent HIV-1 provirus containing *GFP* gene in place of *nef* ^13,24–27^. We found that both RVX-208 and PFI-1 robustly increased the level of GFP expression. 75% GFP-expressing cells were detected at 100 μM of RVX-208 and 89% GFP-expressing cells were determined at 10 μM of PFI-1 in C11 culture models at 72 hours. JQ1 was done as a positive control, which has been widely reported to reactivate latent HIV-1^14,15,28^. Only a small number of cells expressed GFP in DMSO treated group (Fig. 1B). Furthermore, we found that the effects of the two BET inhibitors on reactivation were dose- and time-dependent (Fig. 2A,B). Cytotoxicity measurements under similar conditions demonstrated that RVX-208 and PFI-1 caused minimal cellular toxicity and had minor effects on cell proliferation (Supplementary Fig. 1A,B).

In addition to C11 cells, the two BET inhibitors also can induce latent HIV-1 expression as a function of dose and time (Fig. 2C,D) in the presence of minimal cellular toxicity (Supplementary Fig. 1C,D) in A10.6 cells, another Jurkat-based post-integrative latency model developed in Eric Verdin’s laboratory^29^. In summary, these data show that both RVX-208 and PFI-1 are effective in inducing the expression of latent HIV-1 in Jurkat T cell culture models *in vitro*.

### RVX-208 and PFI-1 activate latent HIV-1 *ex vivo* in resting CD4+ T cells from virally suppressed patients

**Figure 3:**
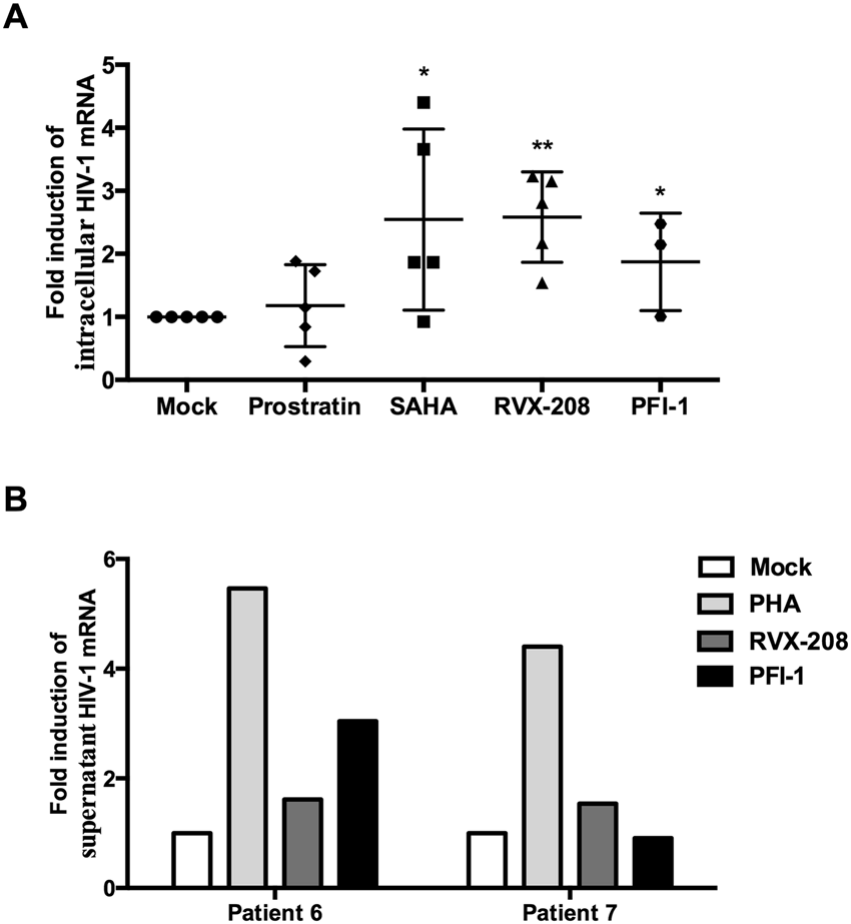
*Ex vivo* activation of HIV-1 expression by RVX-208 and PFI-1. (**A**) Resting CD4+ T cells were isolated from virally suppressed HIV-1-infected patients and pulse-treated with prostratin (1 μM), SAHA (0.5 μM), RVX-208 (50 μM) or PFI-1 (5 μM) for 18 hours. Cell-associated total RNA was extracted, HIV-1 RNA levels were quantified using RT-qPCR for the Poly A region, and fold increase was determined relative to DMSO control. *p < 0.05, **p < 0.01. (**B**) Resting CD4+ T cells isolated from another two HIV-1 patients were treated with PHA (5 μg/ml), RVX-208 (50 μM) or PFI-1 (5 μM) and viral RNA was quantified in cell culture supernatant 18 hours after the addition of drugs. Results are depicted as fold increase in viral RNA relative to control cultures.

Latently infected resting CD4+ T cells represent the major reservoir for HIV-1. We therefore examined whether RVX-208 and PFI-1 treatment also correlated with HIV-1 reactivation in resting CD4+ T cells isolated from patients chronically infected with HIV-1 who were treated with cART. Isolated resting CD4+ T cells were treated with LRAs for 18 hours and cell-associated viral RNA levels were analyzed using primers specific for the HIV-1 3′ polyadenylation (poly A) region. After treatment with RVX-208, increase in HIV-1 full-length transcripts was observed in all five donors, four of which showed >2-fold induction. After treatment with PFI-1, HIV-1 transcripts increasing was observed in two of three donors, the two showed >2-fold induction. SAHA treatment induced an increase in four of five donors, two showed >2-fold induction. Prostratin only increased HIV-1 transcription in three of five donors, one showed a 1.7 fold and another showed a 1.9 fold induction (Fig. 3A).

We next examined whether RVX-208 and PFI-1 induce the release of HIV-1 particles from patient-derived resting CD4+ T cells. We used HIV-1 RNA in cell culture supernatants as a marker for virion release from treated cells. Resting CD4+ T cells from two patients were treated RVX-208 or PFI-1 for 18 hours and the level of supernatant HIV-1 RNA were analyzed using primers specific for the HIV-1 gag gene. RVX-208 treatment induced an increase in the supernatant of cell cultures in both two donors, PFI-1 treatment induced an increase in one of the two donors. PHA treatment was conducted as a positive control (Fig. 3B).

Cytotoxicity measurements were performed in PBMCs to determine the effects of the two BET inhibitors on cell viability and cell proliferation. Results indicated that the 50% cytotoxic concentration (CC_50_) of both RVX-208 and PFI-1 were more than 200 μM, higher than the active concentrations (Supplementary Fig. 2). These results certify the potential of RVX-208 and PFI-1 as candidates of LRAs.

### Combined treatments of RVX-208 and PFI-1 with either prostratin or TNFα synergistic activate latent HIV-1

**Figure 4:**
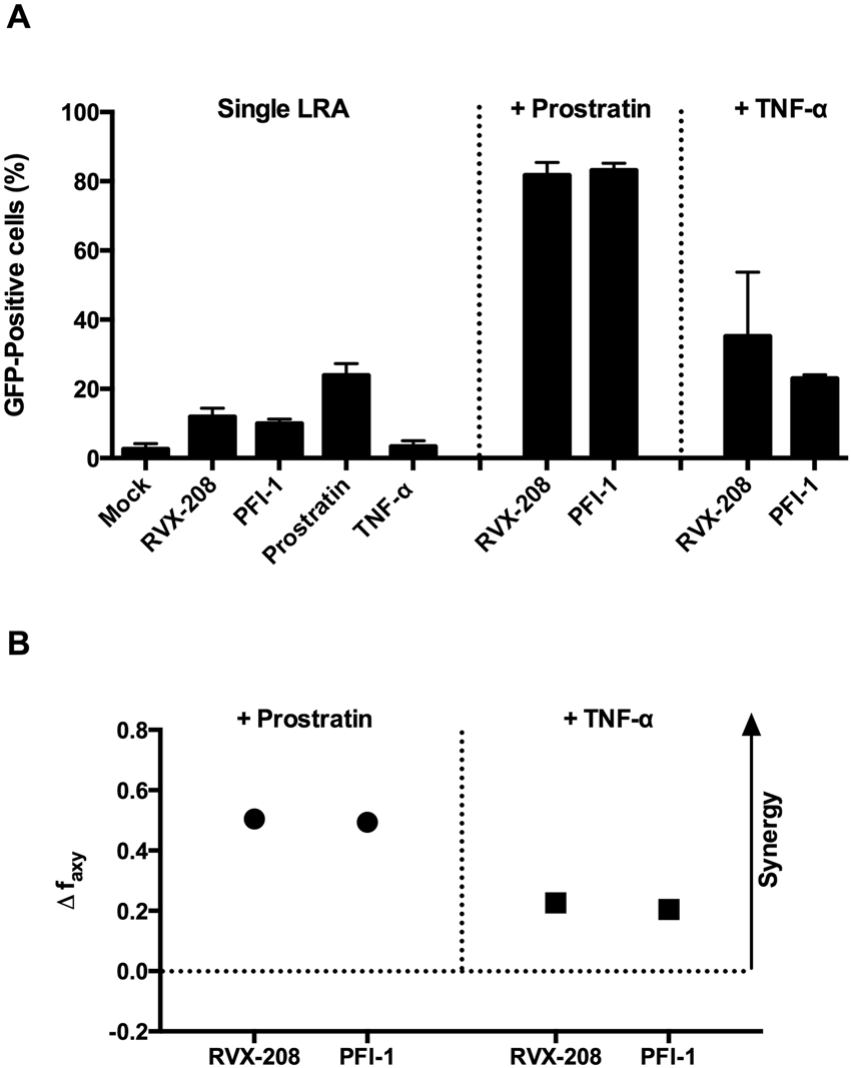
Synergetic activation of HIV-1 expression by BET inhibitors combined with other LRAs. (**A**) C11 cells were incubated with either single BET inhibitors or in combination with prostratin or TNFα for 72 hours. Percentage of GFP-expressing cells was determined by flow cytometry. (**B**) Calculation of synergy for LRA combinations using the Bliss independence model. Data are presented as the difference between the observed and predicted effects. See Methods for more details.

Combinations of mechanistically distinct LRAs may be necessary to overcome the multiple mechanisms governing HIV-1 latency. We therefore measured the synergism of RVX-208 and PFI-1 with other LRAs in latently infected C11 cell culture models. C11 cells were stimulated with either single BET inhibitors, or in combination with the protein kinase C (PKC) activator prostratin (0.2 μM) and the proinflammatory cytokine TNFα (10 ng/ml) for 72 hours. Lower concentrations were used in these assays to more obviously observe the combined effect. As shown in Fig. 4A, RVX-208 (5 μM) treatment alone only stimulated 12% GFP expression in C11 cells. When combined with prostratin or TNF-α, the effect of RVX-208 on reactivation became robust and displayed as 82% and 35%, respectively. PFI-1 (0.1 μM) treatment alone induced 10% GFP expression, and the effect of PFI-1 became 83% and 23% when in combination with prostratin and TNF-α respectively (Fig. 4A). Cytotoxicity measurements demonstrated that there were no effects on cell viability and proliferation when the two BET inhibitors were combined with other LRAs. (Supplementary Fig. 3).

To quantitate whether these combined effects meet criteria for drug synergy, the experimentally observed combined effects was compared to the effects predicted under the Bliss independence model for combined drug effects^30,31^. Effects of combinations that are greater than the idealized Bliss independence prediction imply synergy. We found that the two BET inhibitors synergize significantly with prostratin and TNF-α (Fig. 4B).

### RVX-208 and PFI-1 neither induce global activation of immune cells nor increase cell surface expression of HIV-1 receptors

**Figure 5:**
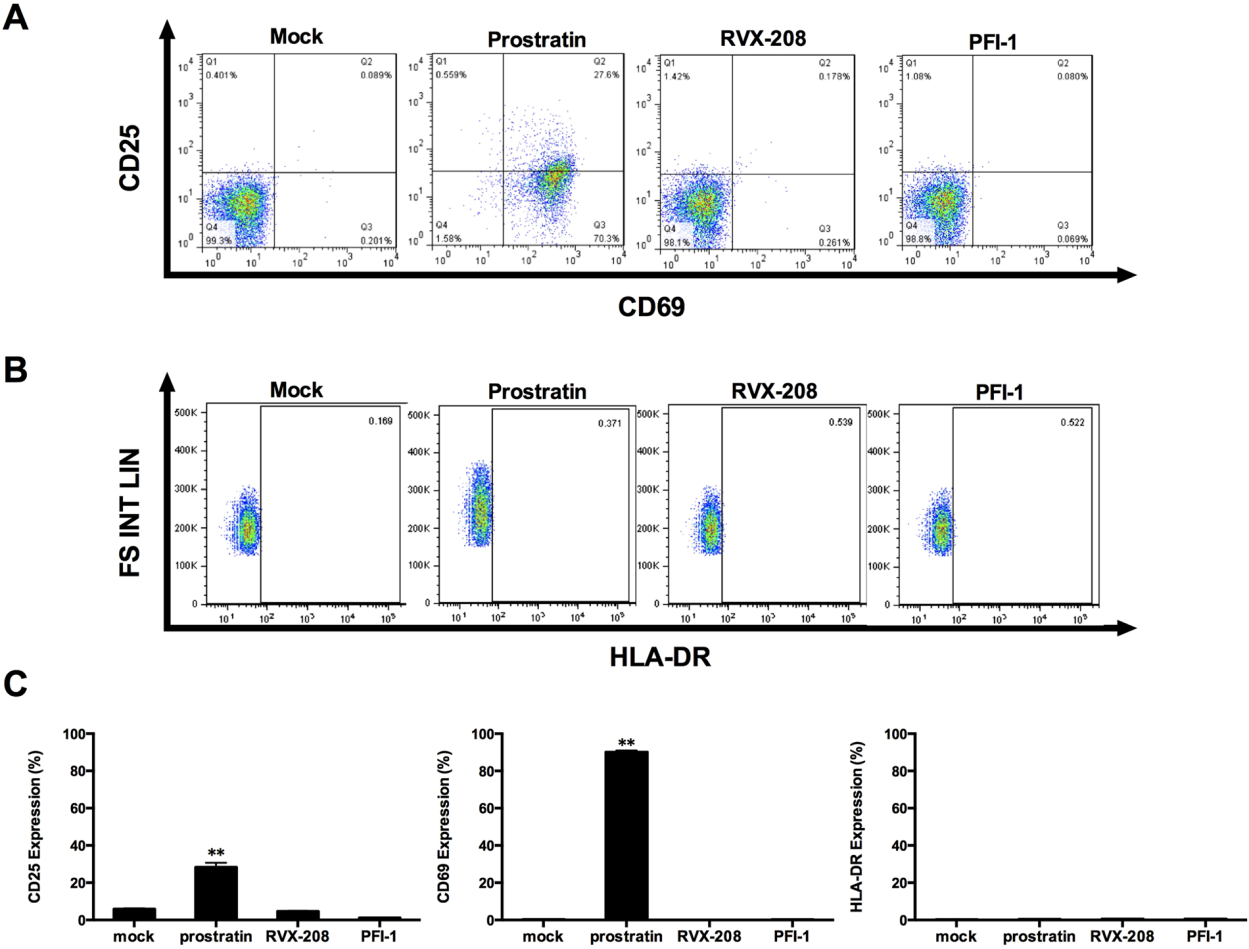
RVX-208 and PFI-1 do not induce global activation of CD4+ T cells. (**A** and **B**) CD4+ T cells isolated from healthy donors were treated with prostratin (1 μM), RVX-208 (50 μM) or PFI-1 (5 μM) and stained for surface markers 72 hours after the treatment initiation. Fractions of CD25, CD69 (**A**) and HLA-DR (**B**) positive cells were analyzed by flow cytometry. (**C**) The percentage of CD25, CD69 and HLA-DR positive CD4+ T cells was calculated. **p < 0.01.

LRAs suitable for therapeutic use should not induce nonspecific immune activation. To determine whether RVX-208 and PFI-1 led to the cellular activation, primary CD4+ T cells isolated from healthy individuals were treated with RVX-208, PFI-1 or prostratin for 72 hours. The activation status was assessed by flow cytometry analysis of the cell surface activation markers including CD25, CD69 and HLA-DR. We observed that RVX-208 treatment did not cause any upregulation of T cell activation markers. Conversely, prostratin treatment robustly induced the surface expression of CD25 and CD69 in CD4+ T cells, which is consistent with previously published results^32^ (Fig. 5). Furthermore, we assessed the CD4+ T cells apoptosis by flow cytometry with Annexin V and PI staining, which could recognize the early and late apoptotic cells respectively, and found the two BET inhibitors make no effect on cell apoptosis (Supplementary Fig. 4).

**Figure 6:**
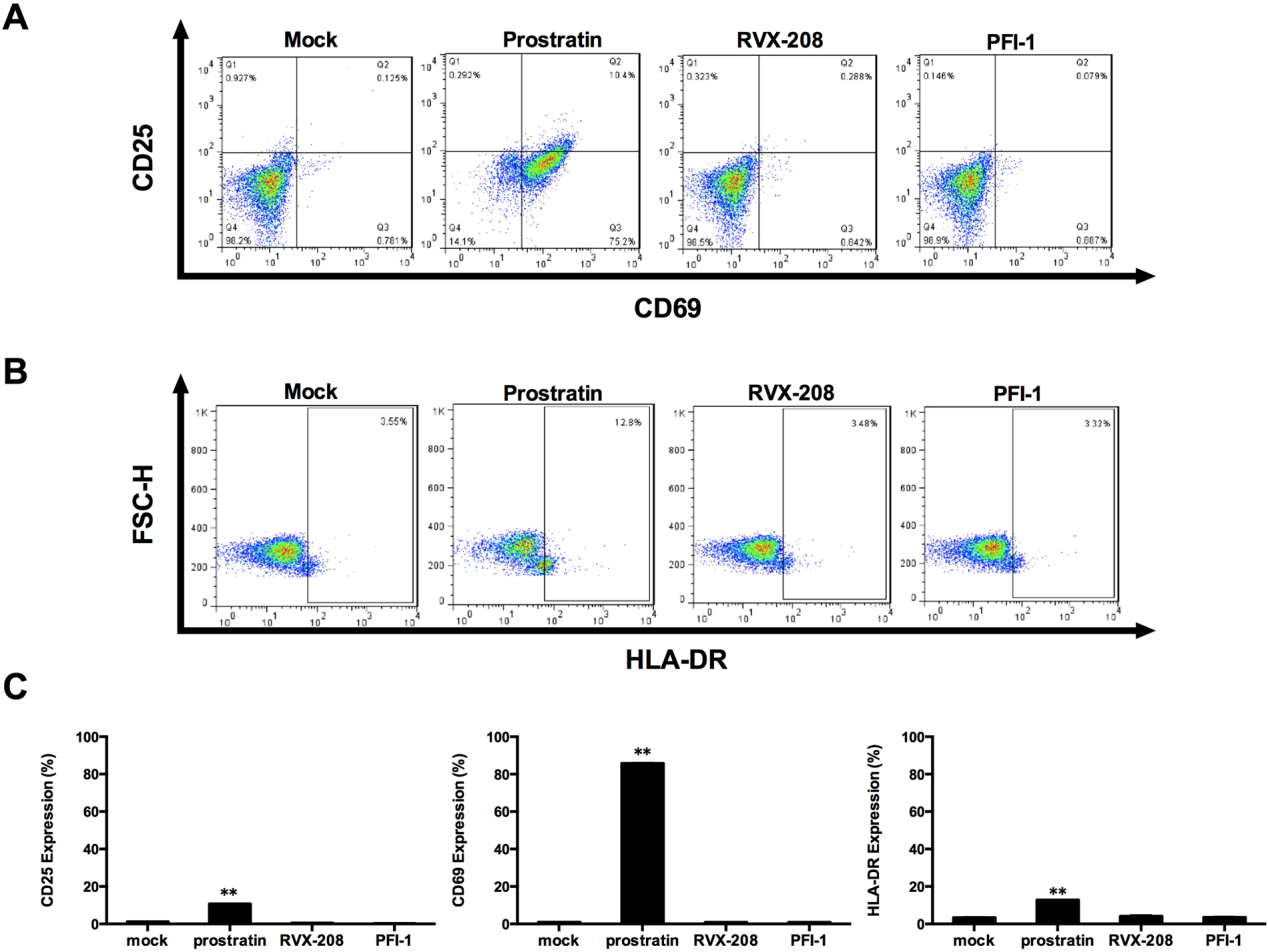
RVX-208 and PFI-1 do not induce global activation of CD8+ T cells. (**A** and **B**) CD8+ T cells isolated from healthy donors were treated with prostratin (1 μM), RVX-208 (50 μM) or PFI-1 (5 μM) and stained for surface markers 72 hours after the treatment initiation. Fractions of CD25, CD69 (**A**) and HLA-DR (**B**) positive cells were analyzed by flow cytometry. (**C**) The percentage of CD25, CD69 and HLA-DR positive CD8+ T cells was calculated. **p < 0.01.

CD8+ T cells might be important for clearing cells with reactivated virus, especially the HIV-1 specific response. We therefore isolated CD8+ T cells and examined the effects of RVX-208 and PFI-1 on activation and proliferation of them. We observed the same phenomenon as in CD4+ T cells that the two compounds did not induce the activation of CD8+ T cells (Fig. 6) and made no influence on cells viability and proliferation (Supplementary Fig. 5).

**Figure 7:**
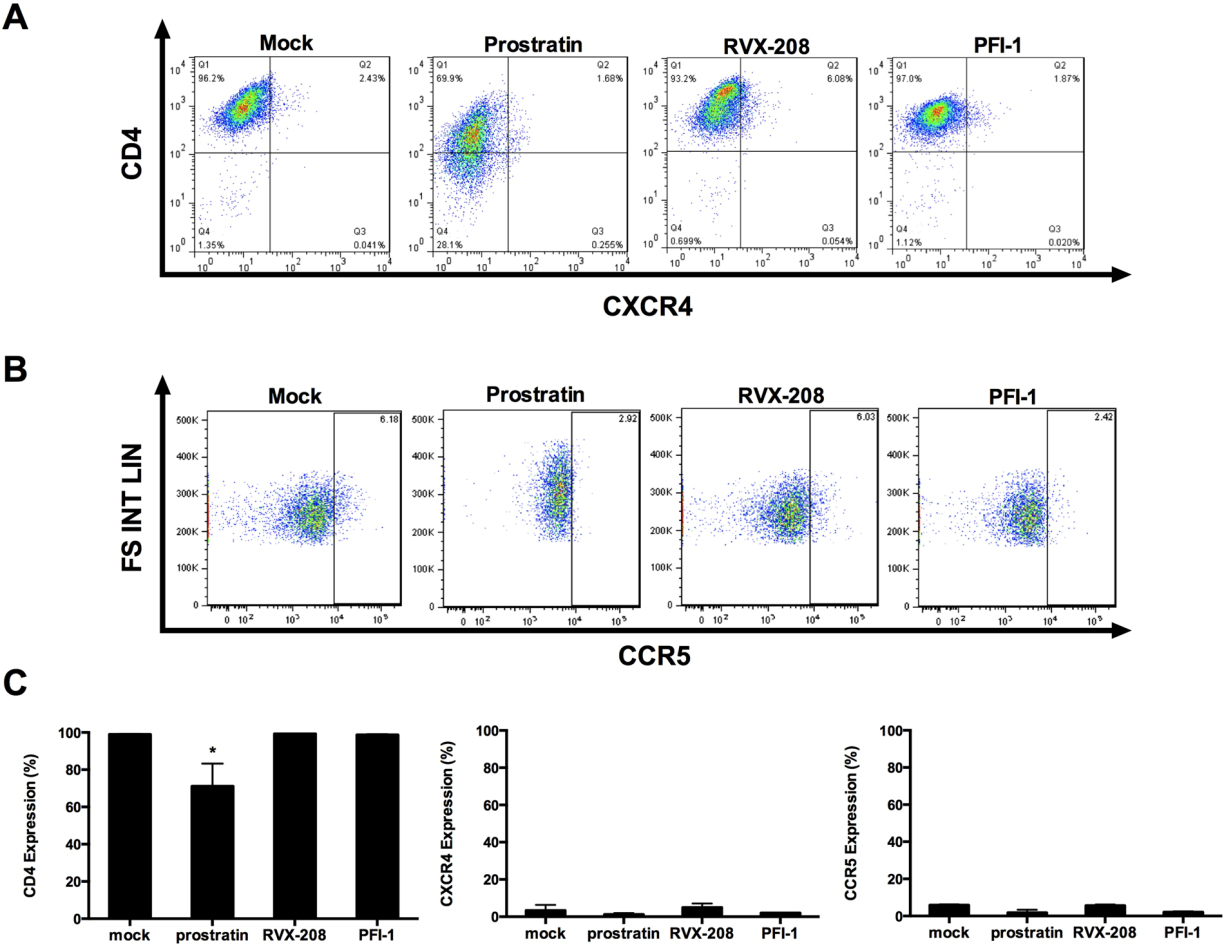
RVX-208 and PFI-1 do not up-modulate the expression of CD4, CXCR4 and CCR5 of CD4+ T cells. (**A** and **B**) CD4+ T cells isolated from healthy donors were treated with prostratin (1 μM), RVX-208 (50 μM) or PFI-1 (5 μM) and stained for surface markers 72 hours after the treatment initiation. Fractions of CD4, CXCR4 (**A**) and CCR5 (**B**) positive cells were analyzed by flow cytometry. (**C**) The percentage of CD4, CXCR4 and CCR5 positive cells was calculated. *p < 0.05.

Drug-mediated change of HIV-1 receptors surface expression is an important factor for *de novo* HIV-1 infection. Prostratin was previously shown to exhibit anti-viral activity by decreasing surface expression of CD4 receptor^32^. We therefore assessed the expression of viral receptor CD4 and co-receptors CXCR4 and CCR5 in primary CD4+ T cells following RVX-208 or PFI-1 treatment. We demonstrated that both RVX-208 and PFI-1 treatments had no effect on the CD4, CXCR4 and CCR5 surface expression, suggesting that RVX-208 and PFI-1 do not increase the possibility of *de novo* HIV-1 infection. Prostratin treatment reduced the cell surface expression of CD4 receptor, which is in agreement with previously published results (Fig. 7).

In conclusion, neither global activation of CD4+ and CD8+ _T cells nor the expression of viral receptors was induced by RVX-208 and PFI-1 treatments, suggesting that these two BET inhibitors may be reliable LRA candidates for further evaluation.

### HIV-1 activation by RVX-208 and PFI-1 correlate with the viral protein Tat

**Figure 8:**
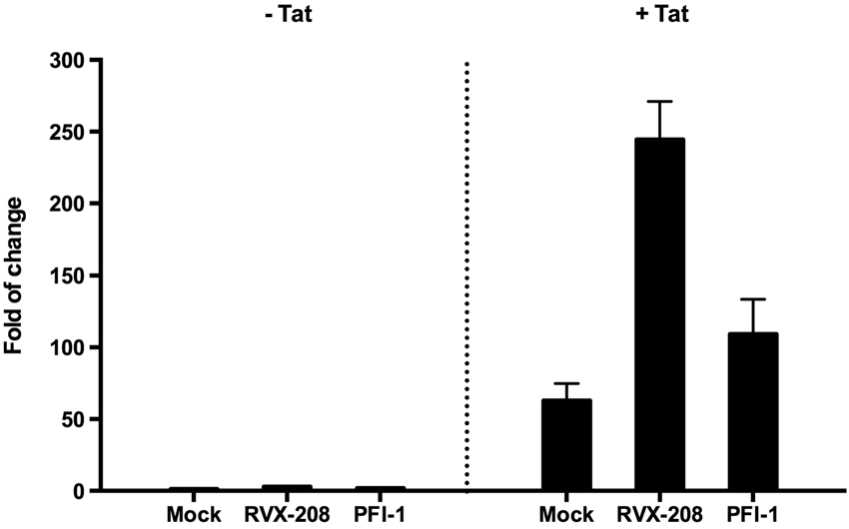
The role of Tat in RVX-208 and PFI-1-mediated activation of latent HIV-1. TZMbl cells were transfected with control or Tat expressing vector, and then received RVX-208 (50 μM) or PFI-1 (5 μM) treatment. Lysates were examined for luciferase activity and depicted as fold increase relative to control.

HIV-1 transcriptional transactivator (Tat) is essential for the synthesis of full-length transcripts from the integrated viral genome by RNA polymerase II (RNAP II). The viral Tat recruits a cellular kinase complex termed P-TEFb to the transactivator RNA (TAR) at the 5′-end of the nascent HIV-1 transcript^33,34^. Considered the important role for Tat in supporting HIV-1 expression and replication, we determined whether BET inhibitors’ reactivation relies on Tat. We used TZMbl cell line, which contains an integrated copy of the HIV-1 LTR with a Luciferase reporter protein. Tat transactivation assays can be readily performed in it by transfection of a Tat expression plasmid and measurement of Luciferase expression from the integrated provirus^34^. We found that the two BET inhibitors only slightly increased the LTR-driven luciferase expression in the absence of Tat (2.7-fold and 1.7-fold). However, when Tat was expressed, RVX-208 and PFI-1 activated the HIV-1 LTR much more potently (244-fold and 109-fold). Tat alone activated 63-fold luciferase expression (Fig. 8). This result indicates that the effect of BET inhibitors on activation of latent HIV-1 is potently enhanced in the presence of Tat in Hela-based TZMbl cells.

### RVX-208 and PFI-1 induce CDK9 Thr-186 phosphorylation

**Figure 9:**
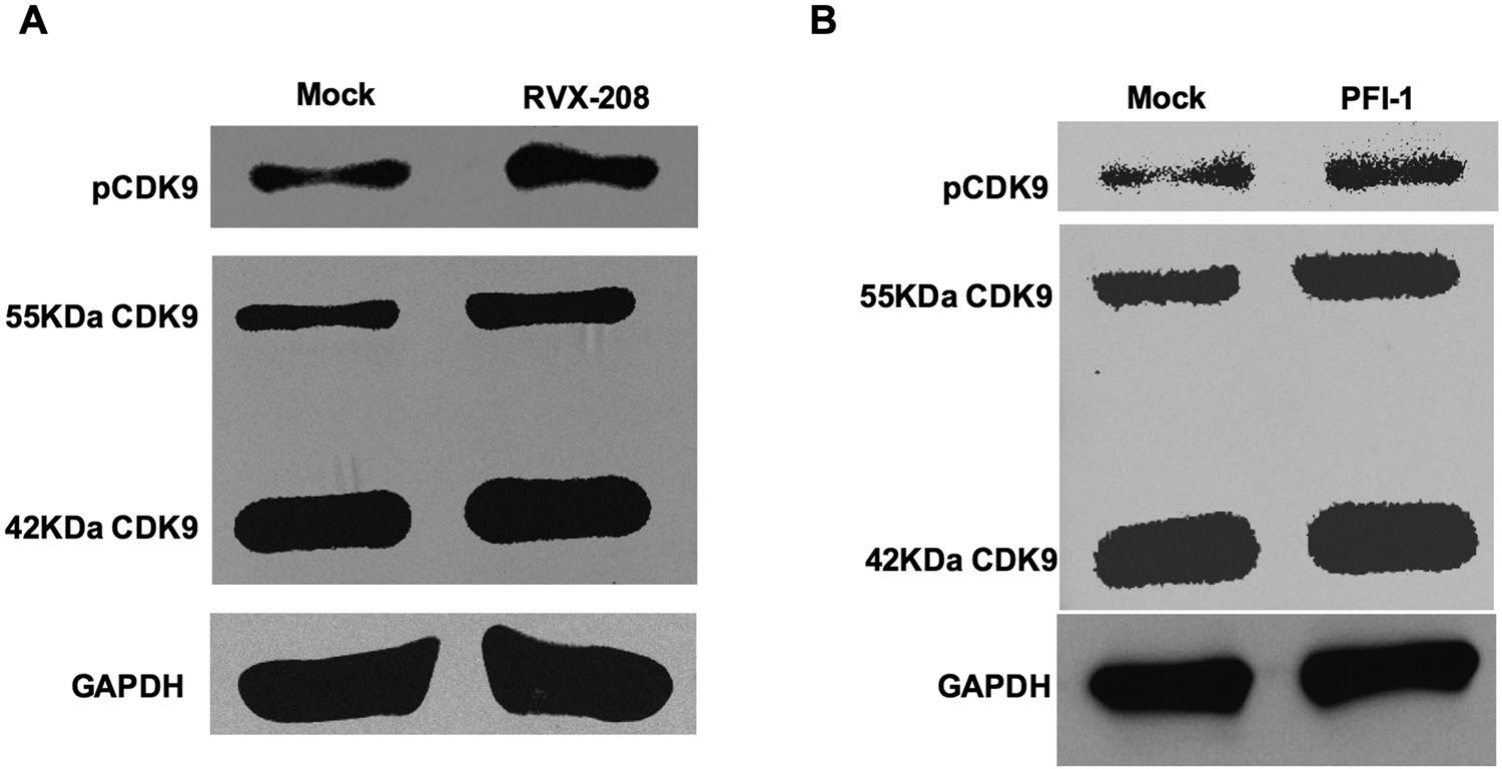
RVX-208 and PFI-1 induce CDK9 Thr-186 phosphorylation. C11 cells were incubated with 50 μM RVX-208 (**A**) or 5 μM PFI-1 (**B**) for 24 hours, cell lysates were analyzed for the expression of CDK9 and pCDK9 in immunoblots. The grouping blots were cropped from different fields. Full-length blots are included in the Supplementary Fig. 6.

P-TEFb stimulates processive transcriptional elongation by phosphorylating the carboxyl terminal domain (CTD) of RNAP II and specific protein subunits of NELF and DSIF, thereby abrogating their inhibition of elongation. In latency cells, P-TEFb function is down-regulated due to low expression levels of subunit cyclin-dependent kinase 9 (CDK9) and repression of CDK9 Thr-186 (T-loop) phosphorylation, a critical posttranslational modification required for kinase activity^35–37^. Because P-TEFb is important for Tat-mediated HIV-1 transcription, we reasoned that the two BET inhibitors reactivation would also likely to up-regulate P-TEFb. We treated C11 cells with them and examined cell lysates for the expression of CDK9 and pCDK9 in immunoblots. There are two isoforms of the CDK9 protein - the major 42KDa CDK9 isoform and the minor 55 KDa isoform^38–40^. We found that there was no change for the expression level of 42KDa CDK9 isoform, but slightly increase for the 55 KDa CDK9 isoform after treatment with RVX-208 or PFI-1. Remarkably, the CDK9 Thr-186 phosphorylation was up-regulated obviously with the help of either the two BET inhibitors. Since the expression level of pCDK9 was not so much as that of CDK9, we only examined the major 42KDa pCDK9 isoform (Fig. 9). Nevertheless, our results indicate that RVX-208 or PFI-1 both promote CDK9 Thr-186 phosphorylation when reversing HIV-1 latency.

## Discussion

Resverlogix is developing RVX-208 to treat cardiovascular disorders, including atherosclerosis, cerebrovascular disease and hypertension. The first-in-class compound could also be beneficial for the treatment of Alzheimer disease. Clinical development is underway in the US for atherosclerosis, acute coronary syndromes and Alzheimer disease^17–20,41–45^. The data from this study show for the first time that RVX-208 acts as a potent activator of latent HIV-1 with effects observed either in diverse Jurkat T latency models or in resting CD4+ T cells isolated from patients chronically infected with HIV-1 who were treated with cART. The widely concentration that RVX-208 reactivated latent HIV-1 in this study is 50 μM. Although it is much higher than the concentration of positive control JQ1, prostratin and SAHA, it is similar to those achieved in HepG2 cells receiving 60 μmol/l RVX-208 followed by assays for apoA-I and HDL-C production. In this study, clinical trial had been conducted in healthy volunteers (given 1 to 20 mg/kg/day of RVX-208) to assess safety, tolerability, and pharmacokinetics, data in humans pointed to beneficial features of RVX-208 that might be useful for treating atherosclerosis^17^. Given the *in vitro* and *ex vivo* results and the established clinical safety profile of RVX-208^44–46^, clinical testing is warranted to assess whether RVX-208 can activate latent HIV-1 and potentially reduce the size of the latent viral reservoir in cART treated HIV-infected patients.

Identified as a BET inhibitor through optimization of a fragment-derived hit, PFI-1 is chemically distinct from previously reported BET inhibitors^21^. The small molecular inhibitor resulted in induction of caspase-dependent apoptosis, differentiation, and in downregulation of the Aurora B kinase in leukemia cells^22,23^. Here, we first report that PFI-1 potently reactivates latent HIV-1 in various post-integration latency Jurkat T cell models *in vitro* and patient-derived resting CD4+ T cells *ex vivo*. Although the concentration of PFI-1 is not lower than that of the positive controls, the CC_50_ of it on PBMCs was more than 200 μM, much higher than the active concentrations (5 μM). These results support further investigations for PFI-1 to antagonize HIV-1 from latency.

Induction of HIV-1 expression from latent reservoirs is being pursued as a component of the reservoir eradication strategy that may ultimately lead to a prolonged drug-free remission or even cure^47–49^. It should be noted that both RVX-208 and PFI-1 reactivated latent HIV-1 to a little degree, which was 2.6-fold and 1.9-fold to the mock control respectively in patient-derived resting CD4+ T cells. It also should be noted that the active concentrations of both RVX-208 and PFI-1 were relatively high. Therefore, additional significant efforts would likely have to be devoted to improving the HIV-1 reactivation effects of the two BET inhibitors. As already suggested, this may potentially be achieved by combining BET inhibitors with other LRAs that work through complementary mechanisms. Here, we documented the synergistic effects on HIV-1 expression between the two BET inhibitors and the PKC activator prostratin or the proinflammatory cytokine TNFα *in vitro*. In future studies, more classes of LRAs should be considered for testing in combination with the two BET inhibitors to identify potential synergies for HIV-1 reactivation *ex vivo* or *in vivo*.

Among the characteristics necessary for clinically testable LRAs, toxicity is a crucial concern. The major problem with present LRAs is the nonspecific induction of many genes and the toxicity caused by the systematic release of cytokines. Ideal LRAs for shock and kill strategies should reverse HIV-1 from latency without causing broad and robust T cell activation^11,50^. Our present data demonstrated that RVX-208 and PFI-1 do not elevate expression levels of CD25, CD69 or HLA-DR, and do not upregulate CD4, CXCR4 or CCR5. We and others have previously reported that BET inhibitors OTX015, JQ1 and UMB-136 exhibit activity to antagonize HIV-1 latency in the absence of global T cell activation^13,16,28^. Generally, given the potency and minimal toxicity, BET inhibitors will be a group of promising candidates for future therapy against reactive latent HIV-1.

HIV-1 replication is dependent on activation of RNAP II by the viral Tat protein. Following transcription initiation from the HIV-1 LTR, RNAP II pauses due to the action of two negative elongation factors, NELF and DSIF, which associate with the RNAP II complex. To activate elongation, Tat recruits P-TEFb to the TAR RNA element at the 5′ end of the nascent viral transcript. Core P-TEFb consists of CDK9 and the regulatory subunit Cyclin T1. P-TEFb stimulates processive transcriptional elongation by phosphorylating the Ser2 residues of the CTD of RNAP II, as well as NELF and DSIF, thereby abrogating their inhibition of elongation^37,51,52^. Thus, viral Tat and cellular P-TEFb levels are key regulators that operate in the context of multiple host factors to influence latency and viral reactivation^28^. Based on the data presented here, we propose that RVX-208 and PFI-1 induce latent HIV-1 expression likely in part through an up-regulation of P-TEFb by increasing CDK9 Thr-186 phosphorylation, and Tat is important in BET inhibitors-mediated reactivation. Also, we need to indicate here that, according to some researchers report, phosphorylation of CDK9 at Thr 186 is not only important for promoting P-TEFb kinase activity but it is also required for P-TEFb to be sequestered into the 7SK snRNP complex^53^. We still don’t know which BET protein RVX-208 and PFI-1 target in inducing HIV-1 expression.

In summary, we initially provide strong evidence that RVX-208 and PFI-1 are attractive potential candidates for HIV-1 cure studies. Our study in combination with previous reports suggests that BET inhibitors represent a group of leading compounds for combating HIV-1 latency for viral eradication and need further study.

## Methods

### Measurement of HIV-1 latency reversal *in vitro*

J-Lat C11 cells^24,26^ (established in our lab) and A10.6 cells^29,54^ (obtained from NIH AIDS Reagent Program) harboring latent, transcriptionally competent HIV-1 provirus that encodes GFP as an indicator of viral activation were cultured in RPMI1640 medium (Gorning) supplemented with 10% fetal bovine serum (FBS) (Gibco), 100 U/mL penicillin and 100 μg/ml streptomycin (Gibco) in a 37 °C incubator containing 5% CO_2_. For *in vitro* reactivation experiments, C11 cells or A10.6 cells were stimulated with RVX-208 (Selleckchem), PFI-1 (Selleckchem), JQ1 (Selleckchem), SAHA (Sigma-Aldrich), prostratin (Sigma-Aldrich) or TNF-α (Sigma-Aldrich) respectively and subjected to determine GFP expression using flow cytometry (BD Calibur).

### Ethics statement

HIV-infected patients were enrolled into the study in Shanghai Public Health Clinical Center and written informed consent was obtained from the patients prior to any study procedures. Clinical and biological characteristics of the patients are listed in Table 1.

**Table 1:** Characteristics of HIV-infected patients receiving cART.

Patients	Gender	Age (years)	CD4+ T cell count (cells/μl)	ART regimen	Duration of therapy (years)	Duration of viral suppression (<40 copies/ml of plasma HIV-1 RNA) (months)
1	Male	51	568	AZT + 3TC + EFV	5	39
2	Male	59	541	TDF + 3TC + LPV/R	2	14
3	Male	31	455	AZT + 3TC + NVP	8	43
4	Male	43	453	AZT + 3TC + EFV	3	27
5	Male	46	388	AZT + 3TC + EFV	4	17
6	Male	35	451	TDF + 3TC + EFV	3	30
7	Male	29	417	AZT + 3TC + EFV	4	40

**Note:** AZT, Zidovudine; 3TC, lamivudine; EFV, efavirnez; TDF, tenofovir; LPV/R, Lopinavir/Ritonavir; NVP, nevirapine.

### Primary cells isolation

These assays were approved by Shanghai Public Health Clinical Center and Shanghai Changhai Hospital. Peripheral blood mononuclear cells (PBMCs) were isolated from fresh whole blood by density gradient centrifugation using Lympholyte®-H Cell Separation Media (Cedarlane Laboratories) as previously described^13,27^. CD4+ T cells were isolated from PBMCs by negative selection using CD4+ T cell Isolation Kit (Miltenyi Biotech). Resting CD4+ T cells were isolated from CD4+ T cells by depletion of cells expressing CD69, CD25 or HLA-DR using CD25-Biotin, CD69-Biotin and HLA-DR-Biotin antibody-coated magnetic beads (Miltenyi Biotech). CD8+ T cells were isolated from PBMCs by positive selection using CD8 MicroBeads (Miltenyi Biotech).

### Measurement of HIV-1 latency reversal *ex vivo*

These assays were modified from Shan *et al*.^55,56^ and approved by Shanghai Public Health Clinical Center. Resting CD4+ T cells (5 × 10^6^) isolated from HIV-infected patients were stimulated with different LRAs for 18 hours under 10 μM T20 treatment. Total RNA was isolated using the ZR-96 Viral RNA Kit (Zymo Research). cDNA was synthesized from the isolated RNA using the the GoScript Reverse Transcription System (Promega) with oligo(dT)_15_ primers. Isolated cDNA was assayed for HIV-1 by RT-PCR using the QuantiFast SYBR Green PCR Kit (QIAGEN) on a Roche LightCycler 480 II machine. Primers used for detecting intracellular RNA were specific for the HIV-1 3′ poly A region: forward (5′-3′) CAGATGCTGCATATAAGCAGCTG and reverse (5′-3′) TTTTTTTTTTTTTTTTTTTTTTTTGAAGCAC. Primers used for detecting supernatant RNA were specific for the HIV-1 gag gene: forward (5′-3′): ATCAATGAGGAAGCTGCAGAA and reverse (5′-3′) GATAGGTGGATTATGTGTCAT. The cycling parameters were as follows: (i) 2 minutes at 50 °C; (ii) 10 minutes at 95 °C; and (iii) 50 cycles at 95 °C for 15 s and then 60 °C for 60 s. Each sample was tested in triplicate, and results were normalized with the human TATA-binding protein (TBP) gene.

### Quantitative analysis of synergy of latency reversing agent combinations

We adapted the Bliss independence model as implemented by Laird *et al*. to test for synergy when BETi was combined with other LRAs^30,31^. For drugs x and y, we used the equation *fa*_*xy,P*_ = *fa*_*x*_ + *fa*_*y*_ − (*fa*_*x*_)*(fa*_*y*_ *)*, where *fa*_*xy,P*_ represents the predicted fraction affected by the combination of drug x and drug y given the observed effects of drug x (*fa*_*x*_) and drug y (*fa*_*y*_) used individually and *fa*_*xy,O*_ = the observed effect when x and y were tested together. Calculation of *fa*_*x*_ as follows: *fa*_*x*_ = *%GFP positive cells with drug x* − *background with DMSO*. With this model, Δ*fa*_*xy*_ = *fa*_*xy,O*_ (the observed fraction affected by the drug combination) − *fa*_*xy,P*_ (the predicted fraction affected by the drug combination) provides an indication of synergy (Δ*fa*_*xy*_ > 0), Bliss independence (Δ*fa*_*xy*_ = 0) or antagonism (Δ*fa*_*xy*_ < 0).

### Cytotoxicity assay

This assay was performed as the manufacturer’s protocol of the Cell Counting Kit-8 (CCK-8) (Dojindo Molecular Technologies) and approved by Shanghai Changhai Hospital. Briefly, approximately 4 × 10^4^ cells per well were treated with different LRAs at the indicated concentrations for the indicated time, and then 10 μl of CCK-8 solution was added to each well. After 4 hours of incubation at 37 °C, the absorbance at 450 nm was measured using a microplate reader.

### Apoptosis Assay

This assay was performed according to the guideline of the Annexin V, FITC Apoptosis Detection Kit (Dojindo Molecular Technologies) and approved by Shanghai Changhai Hospital. Briefly, after incubated with LRAs for 72 hours, primary CD4+ T cells isolated from healthy donors were immunostained with FITC conjunct Annexin V and PI solution for 15 minutes and subjected to flow cytometry analysis.

### Cell activation analysis

This assay was performed according to the guidelines of BD Pharmingen and approved by Shanghai Changhai Hospital, primary CD4+ T cells or CD8+ T cells isolated from blood of healthy donors were stimulated with different LRAs for 72 hours and then stained with CD25-PE, CD69-FITC and HLA-DR-PE antibodies (BD Biosciences) to subject to flow cytometry analysis.

### HIV-1 receptors’ expression analysis

This assay was performed according to the guidelines of BD Pharmingen and approved by Shanghai Changhai Hospital, primary CD4+ T cells isolated from blood of healthy donors were stimulated with different LRAs for 72 hours and then stained with CD4-PE, CXCR4-FITC and CCR5-FITC antibodies (BD Biosciences) to subject to flow cytometry analysis.

### Luciferase assay

Hela-based TZMbl cells containing an integrated HIV-1 LTR-luciferase reporter construct were obtained from NIH AIDS Reagent Program (Dr. John C. Kappes, Dr. Xiaoyun Wu and Tranzyme Inc.) and cultured in Dulbecco’s modified Eagle’s medium (DMSO) (Gorning) supplemented with 10% FBS, 100 U/mL penicillin and 100 μg/ml streptomycin in a 37 °C incubator containing 5% CO_2_. For luciferase assay, TZMbl cells were transfected with Tat or pcDNA 3.1 plasmid using Lipofectamine 3000 Reagent (Invitrogen) 24 hours before the drug treatment. Cells were then treated with LRAs for 48 hours and subjected to determine luciferase activity using Dual-Luciferase Reporter Assay Kit (Promega).

### Western Blotting

As previously described^13,57^, C11 cells were pulse-treated with LRAs for 24 hours and then lysed in buffer containing 50 mM Tris–HCl (pH 8.0), 150 mM NaCl, 2 mM EDTA, 1 mM DTT, 0.1% SDS, 1% Nonidet P-40, 1 mg/ml leupeptin and soybean trypsin inhibitor, 0.5 mM PMSF on ice for 30 minutes. Approximately 50–150 mg of thermally denatured cellular lysates was used for SDS-polyacrylamide gel electrophoresis (SDS-PAGE) immunoblotting analysis. The primary antibodies used were anti-CDK9 and anti-CDK9-pT186 (Cell Signaling Technology). Immunoblotting bands were quantified using the ECL Western blotting system (Santa Cruz Biotechnology).

### Statistical analysis

Data are representative of at least 3 independent experiments, and error bars represent standard deviation. The two-tailed unpaired Student *t* test was adopted to analyze the data sets, where p < 0.05 was considered statistically relevant.

## Electronic supplementary material

Supplementary Dataset
