# Supplementary material for: BET inhibitors RVX-208 and PFI-1 reactivate HIV-1 from latency
Source: Sci Rep. 2017 Nov 30;7:16646. doi: 10.1038/s41598-017-16816-1 (PMC5709369; doi:10.1038/s41598-017-16816-1)
Supplement: Supplementary file 1 — Supplementary Dataset [file 41598_2017_16816_MOESM1_ESM.doc]

***Supplementary Data for***

**BET inhibitors RVX-208 and PFI-1 reactivate HIV-1 from latency**

Panpan Lu^1^, Yinzhong Shen^2^, He Yang^1^, Yanan Wang^1^, Zhengtao Jiang^1^, Xinyi Yang^1^, Yangcheng Zhong^1^, Hanyu Pan^1^, Jianqing Xu^2^, Hongzhou Lu^2^ & Huanzhang Zhu^1^*

^1^State Key Laboratory of Genetic Engineering, Institute of Genetics, School of Life Sciences, Fudan University, Shanghai 200438, China and ^2^Department of Infectious Diseases, and Key Laboratory of Medical Molecular Virology of Ministry of Education/Health, Shanghai Public Health Clinical Center, Fudan University, Shanghai 200433, China.

*To whom correspondence should be addressed. Email: [hzzhu@fudan.edu.cn](mailto:hzzhu@fudan.edu.cn)


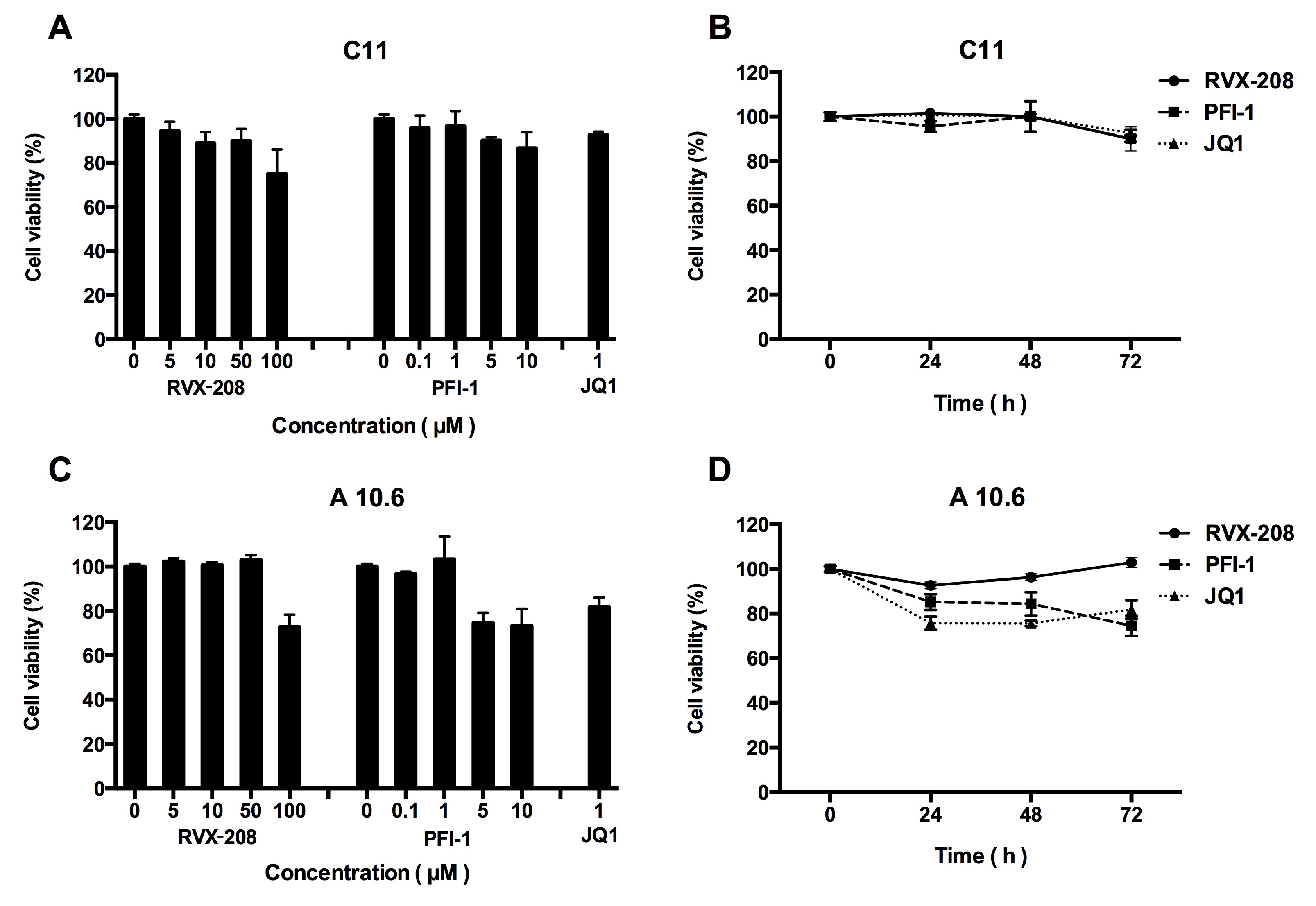


**Supplementary Figure 1. Effects of RVX-208 and PFI-1 on cell viability in latently infected Jurkat T cells.** (A) C11 cells were treated with RVX-208, PFI-1 or JQ1 at the indicated concentrations for 72 hours and cell viability was measured using CCK-8 kit. The division of OD450 between treated and control groups indicate the percentage of cell viability. (B) C11 cells were treated with 50 μM RVX-208, 5 μM PFI-1 or 1 μM JQ1 for the indicated periods of time and cell viability was measured. (C,D) A10.6 cells were treated and analyzed as in (A,B).


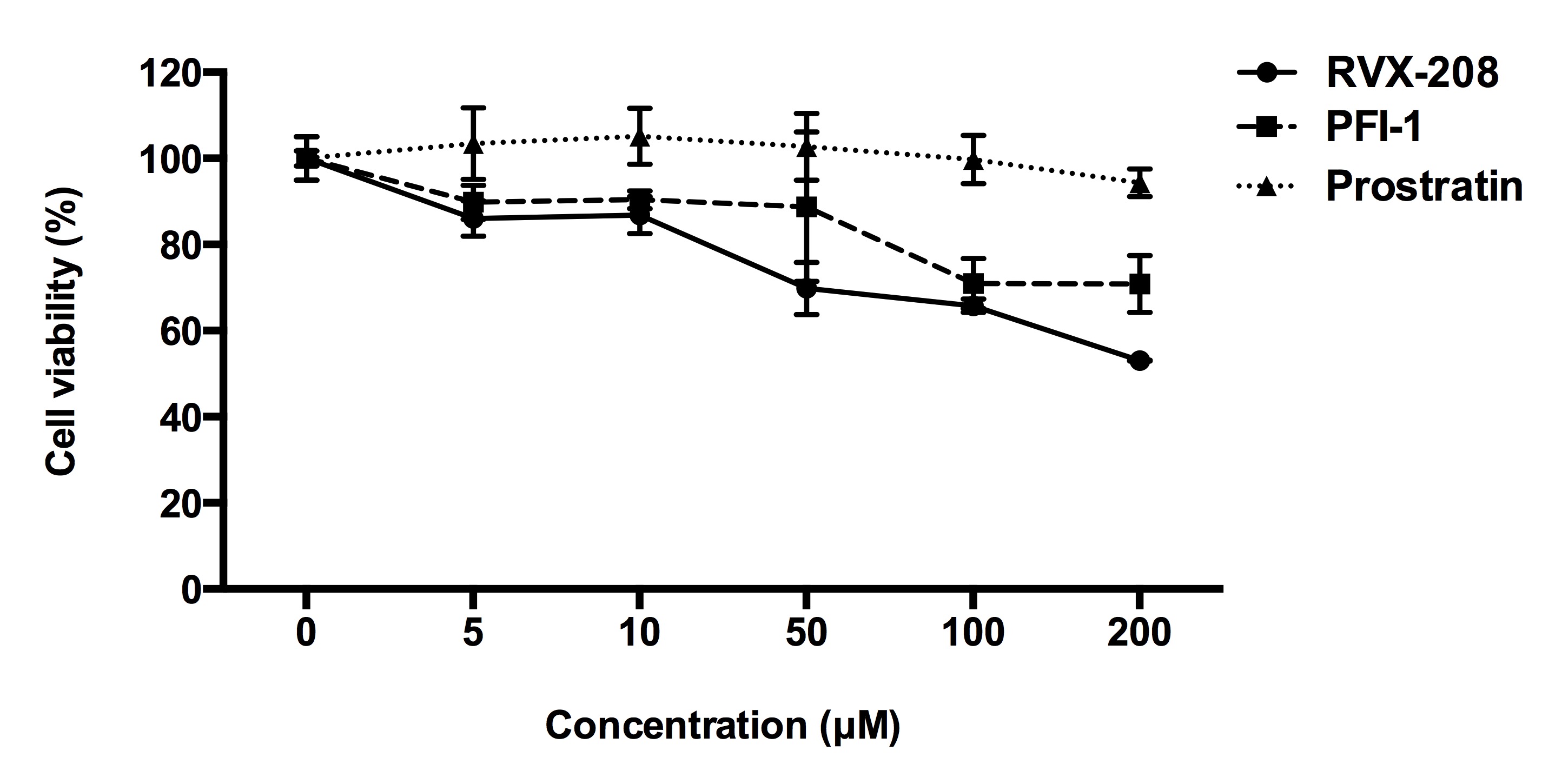


**Supplementary Figure 2. Effects of RVX-208 and PFI-1 on cell viability in PBMCs.** PBMCs from HIV-1-negative donors were treated with RVX-208, PFI-1 or prostratin at the indicated concentrations for 72 hours and cell viability was measured using CCK-8 kit. The division of OD450 between different drug concentrations indicated the percentage of cell viability.


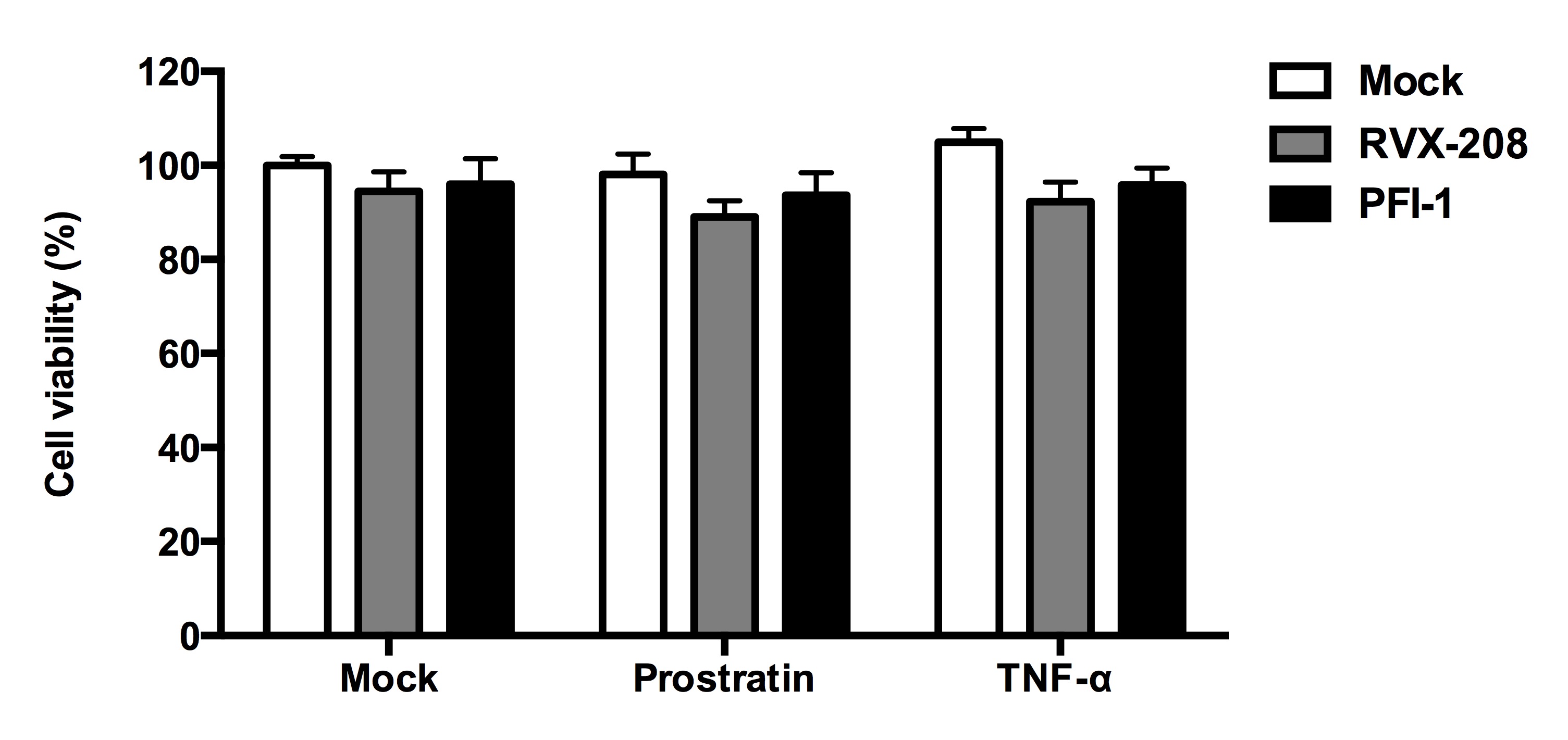


**Supplementary Figure 3. Effects of RVX-208 and PFI-1 in combination with other LRAs on cell viability.** C11 cells were treated with either single BET inhibitors or in combination with prostratin or TNFα for 72 hours and cell viability was measured using CCK-8 kit. The division of OD450 between different drug concentrations indicated the percentage of cell viability.


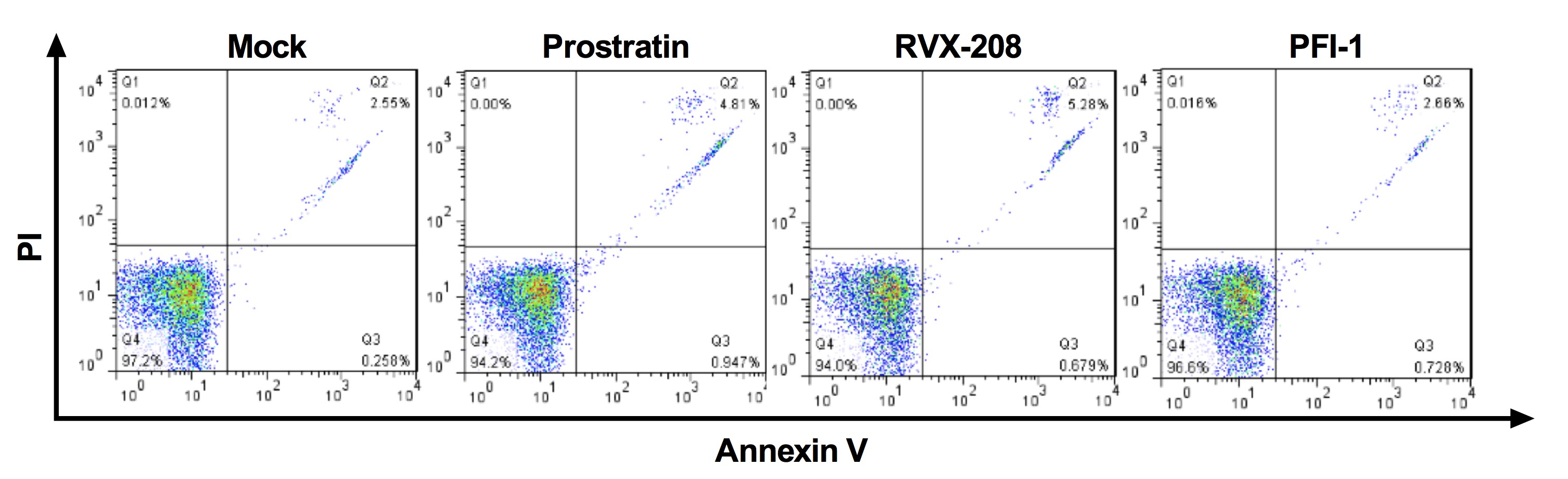


**Supplementary Figure 4. Effects of RVX-208 and PFI-1 on cell apoptosis in CD4+ T cells.** CD4+ T cells isolated from the peripheral blood of healthy donors were treated with prostratin (1 μM), RVX-208 (50 μM) or PFI-1 (5 μM) for 72 hours. Cell apoptosis was determined by Annexin V and PI staining using flow cytometry analysis.


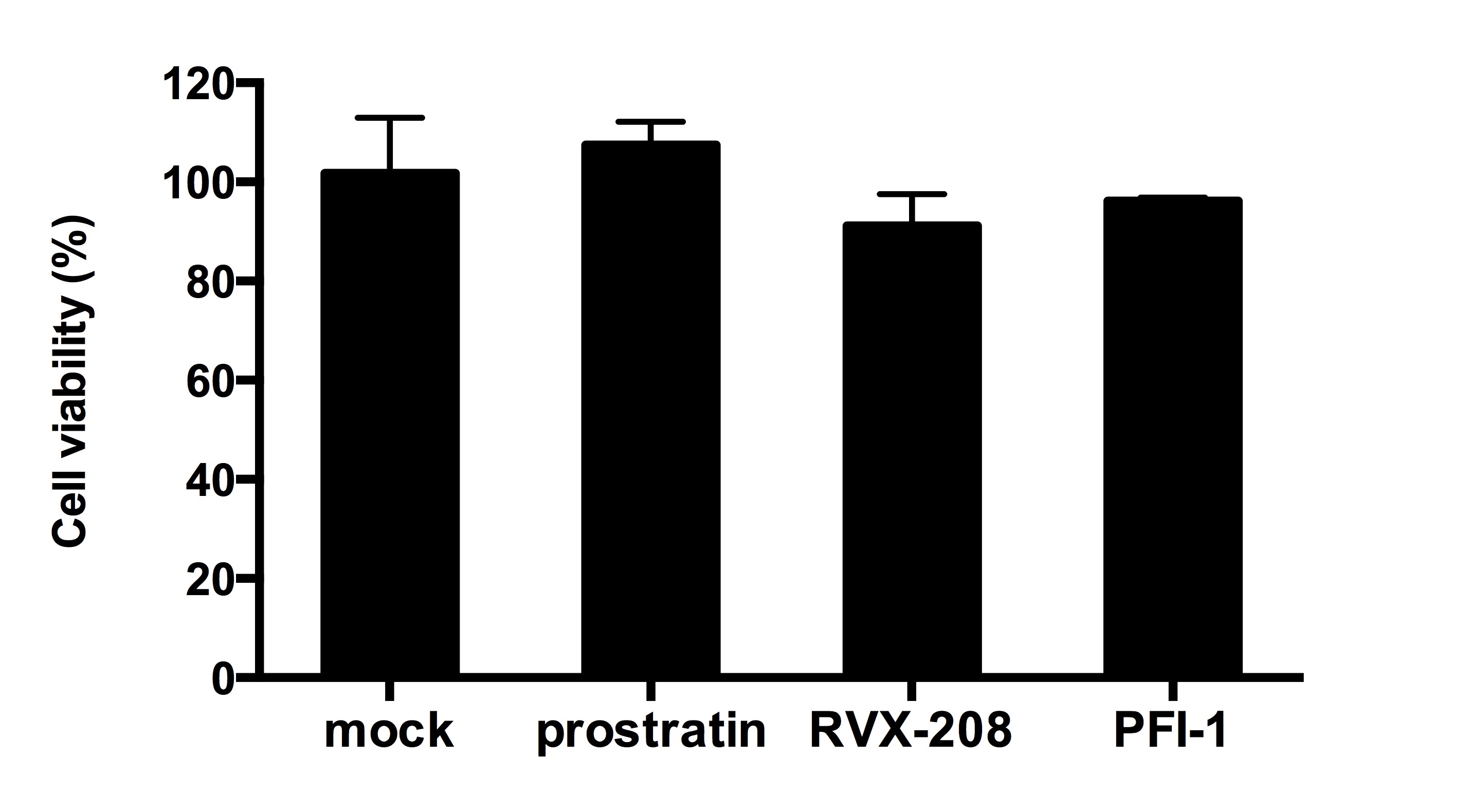


**Supplementary Figure 5. Effects of RVX-208 and PFI-1 on cell viability in CD8+ T cells.** CD8+ T cells isolated from the peripheral blood of healthy donors were treated with prostratin (1 μM), RVX-208 (50 μM) or PFI-1 (5 μM) for 72 hours and cell viability was measured using CCK-8 kit. The division of OD450 between different drugs indicated the percentage of cell viability.


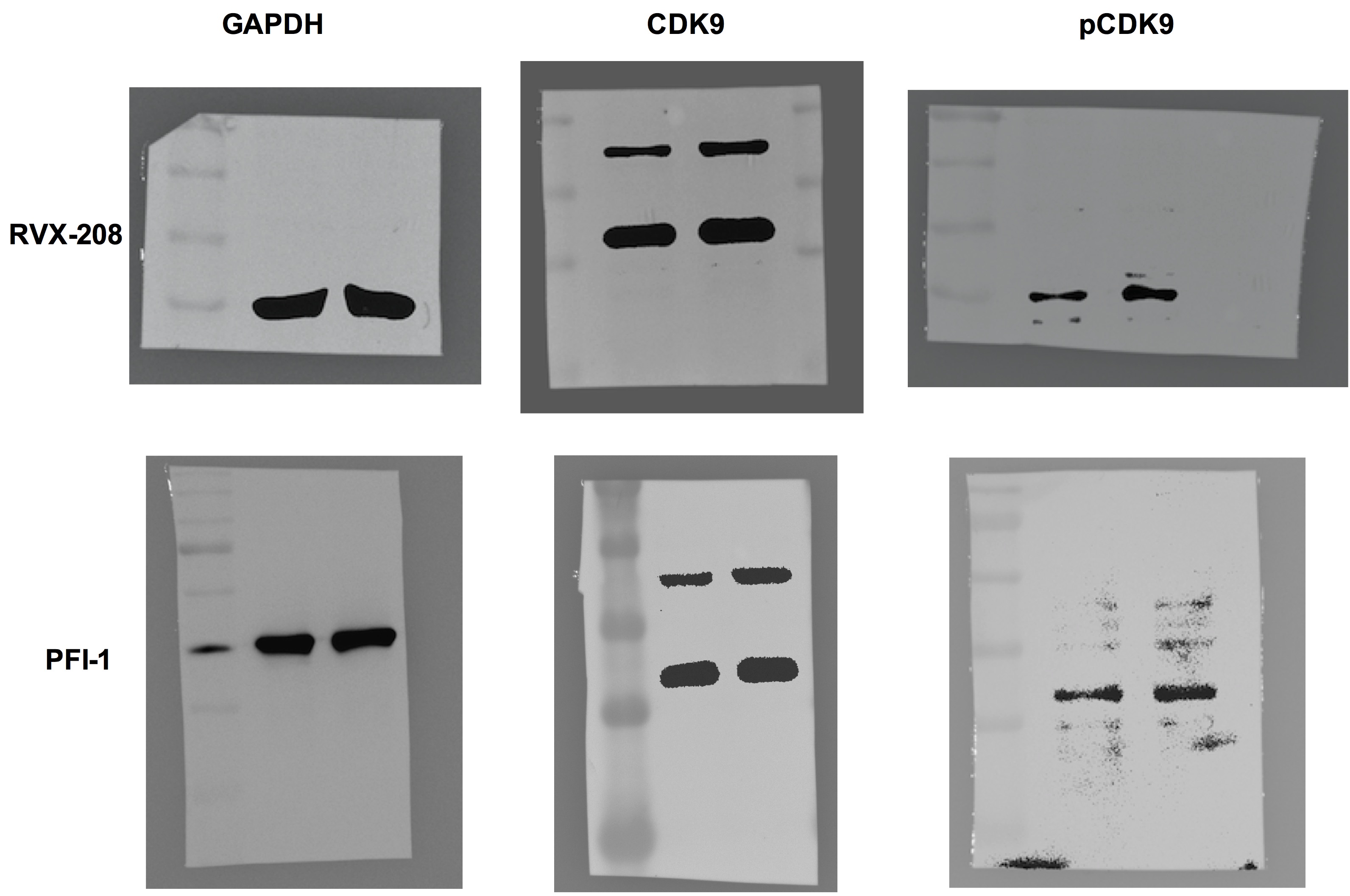


**Supplementary Figure 6. Full-length blots of the Figure 9.**
